# Stathmin 2 is a potential treatment target for TDP-43 proteinopathy in amyotrophic lateral sclerosis

**DOI:** 10.1186/s40035-024-00413-0

**Published:** 2024-04-11

**Authors:** Yunqing Liu, Dejun Yan, Lin Yang, Xian Chen, Chun Hu, Meilan Chen

**Affiliations:** 1 Key Laboratory of Brain, Cognition and Education Sciences, South China Normal University, Ministry of Education, Guangzhou, China; 2 Institute for Brain Research and Rehabilitation, South China Normal University, Guangzhou, 510631 China; 3 Guangdong Second Provincial General Hospital, Guangzhou, 510317 China; 4 Department of Anesthesiology, the Affiliated Panyu Central Hospital of Guangzhou Medical University, Guangzhou, China; 5 Rehabilitation Medicine Institute of Panyu District, Guangzhou, 511499 China

## Main text

Amyotrophic lateral sclerosis (ALS) is a neurodegenerative disease characterized by the selective loss of motor neurons (MNs), resulting in progressive disability and mortality with a rapid course. Current approaches such as multidisciplinary care, disease-modifying therapies, pulmonary intervention, and dietary and nutritional intervention can only slow ALS progression [1]. It is imperative to dissect the underlying mechanisms and explore novel treatment targets.

Trans-reactive DNA binding protein 43 KD (TDP-43) is a main component of abnormal cytoplasmic protein deposits observed in ~ 97% of ALS patients, and its presence is considered a pathological hallmark of ALS regardless of the disease onset. Physiologically, TDP-43 is a multifunctional protein that predominantly localizes to the nucleus, where it binds to GU-rich sequences for selective splicing. It also shuttles to the cytoplasm to generate ribonucleoprotein transport/stress granules and control translation. However, abnormal modifications of TDP-43 reduce its functional level in the nucleus and promotes the formation of cytoplasmic inclusions in MNs, inducing neurotoxic effects known as TDP-43 proteinopathy.

Initial efforts were dedicated to analyzing the binding sites of TDP-43 in mouse and human brains, showing that TDP-43 could target approximately 1000 mRNAs, a large portion being glial RNAs, providing limited insights into neuronal targets. The following study established a method for inducing human embryonic stem cells to differentiate into human MNs (hMNs), providing a more reliable model for investigating disease stimuli and therapeutic strategies [2]. With induced hMNs, Klim et al. [3] revealed that the expression of stathmin-2 (STMN2) was significantly reduced upon TDP-43 depletion. Similar results have been observed in patient-derived MNs and postmortem patient spinal cords harboring TDP-43 mislocalization [4]. Mechanistically, functional TDP-43 binds directly to *STMN2* pre-mRNA to maintain normal splicing. Pathological TDP-43 drives premature polyadenylation and cryptic splicing in the first intron of *STMN2* pre-mRNA, leading to the production of a nonfunctional mRNA [4]. Reduction of TDP-43 or STMN2 in iPSC-derived MNs inhibited axonal regeneration after induced damage. Notably, restoration/stabilization of STMN2 rescued neurite outgrowth and axon regeneration in the absence of TDP-43 [3, 4].

STMN2 belongs to the conserved Stathmin family. It can depolymerize microtubules via unclear mechanisms and is specifically expressed in the nervous system for axonal development and maintenance (see details in [5]). A moderate level of STMN2 stimulates neurite outgrowth by modulating microtubule dynamics, whereas excessive or reduced levels of STMN2 cause growth cone collapse or suppress neurite outgrowth in neurons. In cultured sensory neurons from dorsal root ganglion (DRG) subjected to axotomy, Stmn2 was elevated in regenerating growth cones. Downregulation of Stmn2 accelerated axon fragmentation, whereas experimental rescue of the Stmn2 level delayed axonal degeneration [6]. Similarly, loss of *Stai*, a homolog of *STMN2* in *Drosophila*, leads to neuromuscular junction (NMJ) degeneration and motor axon retraction [7, 8]. Recently, Krus et al. generated both constitutive and conditional *Stmn2* knockout mice and reported that Stmn2 is required for motor and sensory system function [9]. Constitutive *Stmn2* knockout (*Stmn2*^−/−^) induces severe motor and sensory neuropathy, including decreased compound muscle action potentials, NMJ denervation, and reduced nerve fiber density. Importantly, *Stmn2*^−/−^ mice predominantly exhibit degeneration of fast-fatigable motor units, similar to that observed in ALS patients. Loss of Stmn2 specifically in MNs recapitulates the NMJ pathology found in *Stmn2*^−/−^ mice [9]. The authors further studied Stmn2^+/−^ mice, which mimic the partial loss of STMN2 in ALS patients and exhibit selective motor neuropathy. Like *Stmn2*^−/−^ mice, the *Stmn2*^+/−^ heterozygous mice behave normally as young adults but show motor weakness by 1 year of age [9]. This progressive motor neuropathy is also a typical clinical symptom of ALS patients. Moreover, adult mice with absence of Stmn-2 exhibit phenotypes comparable to those of ALS patients [10], suggesting that STMN2 is involved in ALS pathology.

Nevertheless, there is emerging evidence of aberrant STMN2 in ALS patients. A noncoding CA repeat in *STMN2* that may affect mRNA processing has been reported to be associated with sporadic ALS in a North American cohort [11]. Moreover, two independent groups detected cryptic exons of *STMN2* in postmortem brain tissues from patients with TDP-43-associated Alzheimer’s disease [12] and C9ORF72 patients who were susceptible to TDP-43 pathology [13]. Consistently, in an unbiased study of single-cell protein expression profiles with human spinal MNs directly sampled from TDP-43 ALS patients, a lower frequency of the STMN2 protein was detected [14]. Via in situ hybridization, they detected a robust decrease in the *STMN2* RNA level in ALS MNs [14]. Importantly, cryptic splicing of *STMN2* was confirmed in TDP-43-depleted human iPSC-derived MNs [15] and iPSC MNs from postmortem sporadic TDP-43 ALS patients [16]. Thus, these findings reveal a strong link between aberrant *STMN2* expression and MN degeneration in ALS and imply that restoring STMN2 levels is a promising therapeutic approach for TDP-43-dependent ALS.

**Fig. 1:**
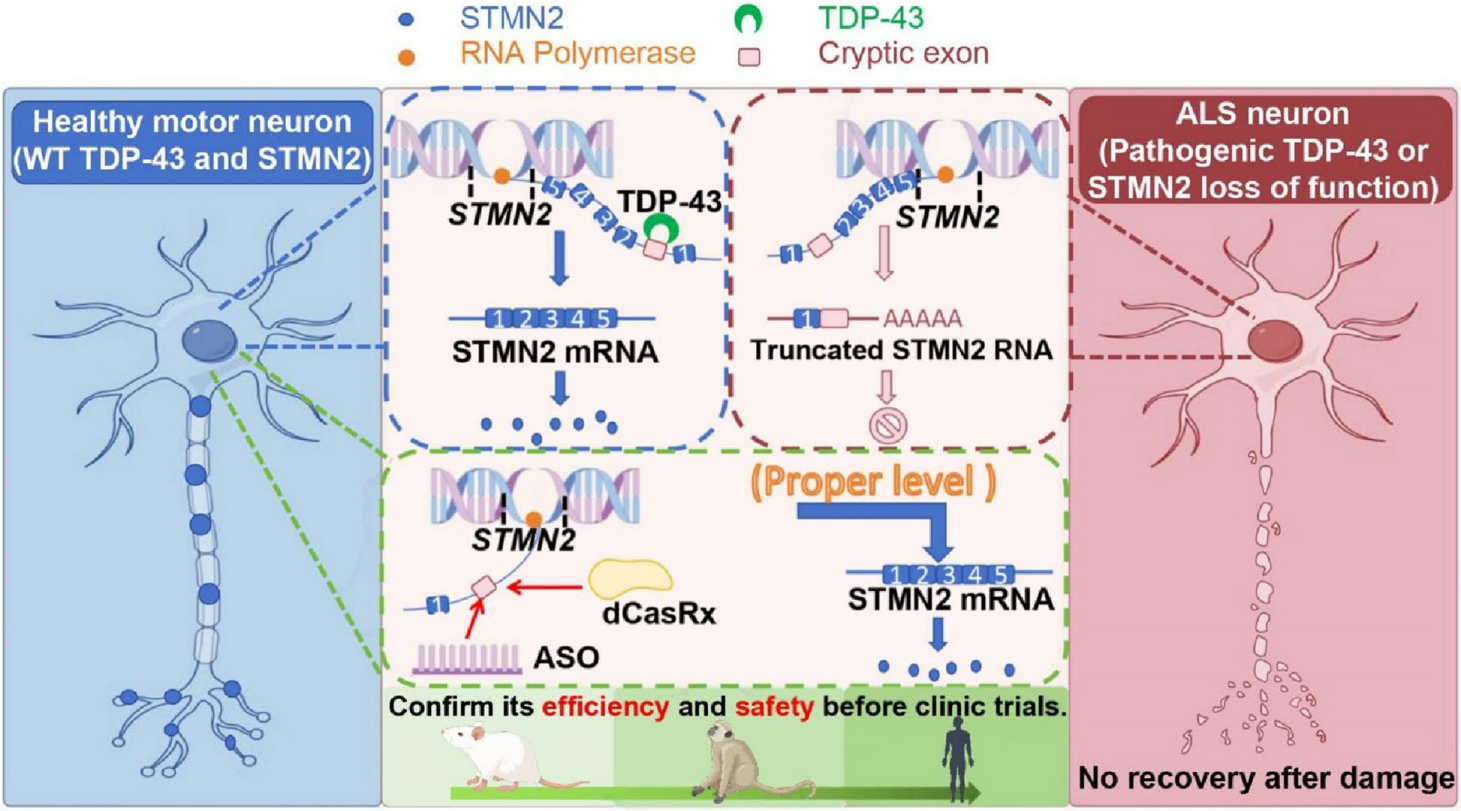
TDP-43 binds directly to *STMN2* pre-mRNA to guarantee normal splicing of *STMN2* mRNA. Pathogenic (reduced) TDP-43 drives premature polyadenylation and aberrant splicing by steric inhibition in the first intron of the STMN2 pre-mRNA, producing a non-functional mRNA. Using dCasRx or antisense oligonucleotides (ASOs) to target the first intron of the *STMN2* pre-mRNA can efficiently restore STMN2 level and axonal regeneration in TDP-43 proteinopathy. Although current studies have provided promising results, animal models are required to confirm the efficiency and safety before clinic trials

To test the effect of correcting *STMN2* pre-mRNA metabolism against TDP-43 proteinopathy, Baughn et al. pioneered this study to elucidate the detailed mechanism by which TDP-43 modulates STMN2 expression. They used CRISPR-Cas9 to clarify that TDP-43 binding to the exon 2a (a region containing a 24-base GU-rich segment between the cryptic splicing site and polyadenylation site in the first intron) of *STMN2* prevents misprocessing by blocking the recognition of cryptic RNA elements [17]. They subsequently substituted the 24-base GU-rich domain with a 19-base segment encoding the bacteriophage MS2 aptamer sequence, an RNA stem-loop structure that can be bound by the MS2 coat protein, thus preventing direct TDP-43 interaction. This substitution resulted in constitutive misprocessing of STMN2 pre-mRNA. Further genome editing analysis revealed that instead of the cryptic polyadenylation site, the cryptic 3’ splice acceptor is essential for initiating *STMN2* pre-mRNA misprocessing. Based on this critical finding, they attempted to suppress cryptic splicing of *STMN2* pre-mRNA by use of dCasRx (the “nuclease-dead” variant of the CRISPR effector RfxCas13d, which retains RNA-binding capability without enzymatic activity) or antisense oligonucleotides (ASOs), which restored STMN2 levels and axonal regeneration in TDP-43-deficient human MNs. Critically, ASOs injected into the cerebral spinal fluid of mice containing humanized STMN2 with cryptic splice-polyadenylation sequences could restore Stmn2 protein level and axonal regrowth [17] (Fig. 1).

Collectively, these studies indicate that a reduction in STMN2 is a critical biomarker for TDP-43 proteinopathy. Approaches that can restore STMN2 protein level are likely efficient in promoting MN regeneration. However, all current studies lack in vivo examination of functional/behavioral outcomes. Another core issue is how to maintain moderate levels of STMN2 since increased or decreased expression of STMN2 could be a barrier to axonal outgrowth/regeneration during patient treatment. Despite the gap from bench to bedside, STMN2 is a potential therapeutic target for TDP-43 proteinopathy.

## Data Availability

Not applicable.

## Abbreviations

ALS: Amyotrophic lateral sclerosis
ASO: Antisense oligonucleotide
hMN: Human motor neuron
MN: Motor neuron
NMJ: Neuromuscular junction
TDP-43: Trans-reactive DNA binding protein 43 KD
STMN2: Stathmin 2
